# Genomic Dissection of Peduncle Morphology in Barley through Nested Association Mapping

**DOI:** 10.3390/plants10010010

**Published:** 2020-12-23

**Authors:** Sebastian Zahn, Thomas Schmutzer, Klaus Pillen, Andreas Maurer

**Affiliations:** Martin Luther University Halle-Wittenberg, Institute of Agricultural and Nutritional Sciences, Chair of Plant Breeding, Betty-Heimann-Str. 3, 06120 Halle, Germany; sebastian.zahn@landw.uni-halle.de (S.Z.); thomas.schmutzer@landw.uni-halle.de (T.S.); klaus.pillen@landw.uni-halle.de (K.P.)

**Keywords:** barley (*Hordeum vulgare* ssp. *vulgare*), wild barley (*Hordeum vulgare* ssp. *spontaneum*), nested association mapping (NAM), genome-wide association study (GWAS), peduncle morphology

## Abstract

Straw biomass and stability are crucial for stable yields. Moreover, straw harbors the potential to serve as a valuable raw material for bio-economic processes. The peduncle is the top part of the last shoot internode and carries the spike. This study investigates the genetic control of barley peduncle morphology. Therefore, 1411 BC1S3 lines of the nested association mapping (NAM) population “Halle Exotic Barley 25” (HEB-25), generated by crossing the spring barley elite cultivar Barke with an assortment of 25 exotic barley accessions, were used. Applying 50k Illumina Infinium iSelect SNP genotyping yielded new insights and a better understanding of the quantitative trait loci (QTL) involved in controlling the peduncle diameter traits, we found the total thickness of peduncle tissues and the area of the peduncle cross-section. We identified three major QTL regions on chromosomes 2H and 3H mainly impacting the traits. Remarkably, the exotic allele at the QTL on chromosome 3H improved all three traits investigated in this work. Introgressing this QTL in elite cultivars might facilitate to adjust peduncle morphology for improved plant stability or enlarged straw biomass production independent of flowering time and without detrimental effects on grain yield.

## 1. Introduction

In total, 11.592 million tons of barley grains were harvested in Germany in 2019, of which spring barley accounted for 1.829 million tons [1]. The mean grain:straw ratio in current German barley varieties amounts to 1:0.7. As 35% of barley straw can be harvested without negatively impacting the humus accounting [2], 2.84 million tons of barley straw are available as resource on German fields. Therefore, it is a considerable source of biomass. The bio-economic sector uses raw materials containing lignocellulose (like straw) in manifold processes. Lignocellulose forms the major component of plant cell wall and is composed of cellulose, hemicellulose, and lignin [3]. The high occurrence of lignocellulose in plants makes it the most abundant type of terrestrial biomass [4]. Harvesting residues can be processed in lignocellulosic biorefineries to bulk products as biofuels, as well as to high-value products, including biochemical products [5]. As versatile as the applications are, the requirements regarding the raw material are equally diverse. The various possible adjustments to barley straw and potential increase of raw material justify deeper research on the matter.

Growers are focused on a safe grain harvest, requiring stable and healthy straw to secure a robust plant stature. During the Green Revolution, the introduction of semi-dwarfing genes led to shorter and sturdier culms enhancing grain yield and to a more secure harvest due to a lower risk of lodging [6]. Unfortunately, semi-dwarfing genes come with a trade-off for yield-relevant organ size [7]. According to Schnurbusch [8], more basic research on culm traits is needed at this point to solve this issue in the future. 

In barley, vegetative internodes elongate acropetally. The final internode extension pushes the flowering head out of the flag leaf. This emergence is called “heading” (HEA) and happens around anthesis [9]. The right timing for HEA/anthesis is a crucial life decision in all plants [10]. Heading time often decides the suitable adaptation and yield potential of crops [8]. The peduncle is the top part of the last internode to expand and drives out the flowering head. Little is known about the variation and the quantitative trait loci (QTL) regions controlling the barley peduncle. A study in 2014 regarding lodging parameters of a biparental population gave a first insight into this topic [11]. By means of a QTL study conducted with 117 SSR markers, the authors analyzed the traits diameter and wall thickness of six internodes. Our study aims to better understand the QTL regions controlling the barley peduncle morphology with a genome-wide association study (GWAS) using a 50k Illumina Infinium iSelect SNP Array [12] on 2-year field data of the wild barley nested association mapping (NAM) population “Halle Exotic Barley 25” (HEB-25). This population provides a large and diverse panel of wild barley alleles in a cultivated Barke background [13]. In this study, we aim at yielding new insights and a better understanding of the QTL regions involved in controlling the traits peduncle diameter (DIA), total thickness of peduncle tissues (WAL), and the area of the peduncle cross-section (AREA). These traits have not been broadly investigated to date. They are assumed to provide an overview of the potential variation in culm morphology of wild barley. The evaluation on their usefulness for future projects is part of this basic research as they might serve as proxies for evaluating the lignocellulose yield while retaining a stable plant stature.

## 2. Results and Discussion

### 2.1. Descriptive Statistics

**Figure 1:**
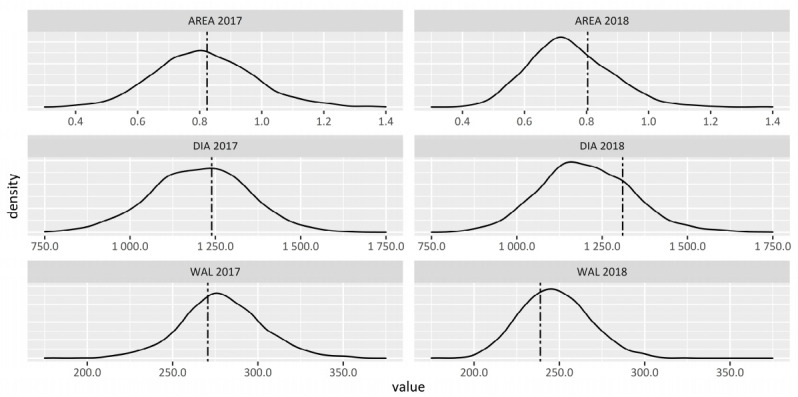
Frequency distribution of measured peduncle morphology traits 2017 and 2018 with highlighted Barke mean (dot dashed line). Traits: Peduncle diameter (DIA [µm]), total thickness of peduncle tissues (WAL [µm]), area of the peduncle cross-section (AREA [mm^2^]); n = 1232 (2017), n = 1343 (2018).

The population showed a broad variation for all traits between genotypes (Figure 1), representing a promising resource for GWAS. The coefficient of variation across all genotypes of the population (CV, Table 1) was approximately in the dimension of other traits successfully investigated in HEB-25 [14]. Whereas DIA had a stable mean of approximately 1.2 mm across years (*t*-test, *p* = 0.788), the values for WAL were significantly (*t*-test, *p* < 0.001) higher in 2017 (279 µm) than in 2018 (247 µm, Table 1). The impact of the factor year on the trait WAL is also reflected in the high estimate of the environmental variance component, while it had no impact on DIA (Table 2). The difference in WAL between years might be explained by changed environmental conditions under naturally occurring drought stress in 2018 (Figure S1). Drought might have directly affected WAL, while DIA, as the more heritable trait, remained unaffected (Table 2). The analysis of broad-sense heritability resulted in H^2^ = 73.3% for DIA, H^2^ = 52.6% for WAL and H^2^ = 67.0% for AREA. Furthermore, it was striking that Barke, the reference elite parent of the population, had an increased DIA and AREA, while it had a thinner WAL compared to the population mean.

### 2.2. Correlations

**Figure 2:**
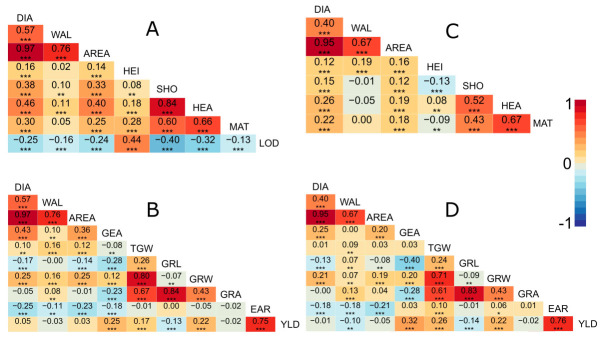
Correlation chart (Pearson correlation coefficients) of peduncle traits with developmental traits 2017 (**A**) and 2018 (**C**) and yield traits 2017 (**B**) and 2018 (**D**). The heatmap coloring indicates the correlation strength. Asterisks indicate the significance level (* *p* < 0.05, ** *p* < 0.01, *** *p* < 0.001). Autocorrelations of traits between years were 0.57 (*p* < 0.001) for DIA, 0.37 (*p* < 0.001) for WAL and 0.51 (*p* < 0.001) for AREA. Abbreviations: HEI: Plant height, SHO: Time to shooting, HEA: Time to heading (flowering time), MAT: Time to maturity, LOD: Lodging, YLD: Grain yield, TGW: Thousand grain weight, GRW: Grain width, GRL: Grain length, GRA: Grain area, GNE: Grain number per ear, EAR: Ears m^−2^.

Previous studies have indicated correlations between lodging, heading, and culm diameter. For instance, in spring wheat, lodging is strongly influenced by the wall thickness at the basal internodes, as well as by the stem diameter, including all internodes and the peduncle. Due to higher correlations at the basal internodes between culm morphology and lodging resistance, researchers have suggested breeding for a bigger diameter and wall thickness at the lower end of the culm [15]. In rice, the culm diameter and heading date are closely linked to pushing resistance. Also, for this crop, the conclusion is that increasing stem diameter would improve the lodging resistance [16]. Following the abovementioned studies, a correlation analysis was performed. The data for growth parameters and yield parameters for the same field trial were taken from [17]. Significant correlations between lodging (LOD), heading (HEA), culm diameter (DIA), and thickness (WAL) were observed (Figure 2A,C), confirming their relationship in barley. In general, DIA was positively correlated with HEA and negatively correlated with LOD. Also, WAL was negatively correlated with LOD, indicating that DIA and WAL might impact lodging resistance concertedly. A study of a biparental barley population pointed out significant correlations (*r* = −0.146; *p* < 0.05) between the wall thickness of the sixth internode and lodging in accordance with our observed correlation, while no significant correlations between diameter and lodging were found. Diameter and wall thickness themselves were correlated in the same manner as in our study [11]. The differences in the correlation findings between diameter and lodging in comparison to our study might be attributed to the different germplasm and the different methods used for the measurements (caliper vs. microscope). In comparison with the above mentioned plant stability studies in wheat and rice, the peduncle data from the present study shows comparable results [15,16]. This might indicate that peduncle data serve as proxy to evaluate the general plant stability. Probably, due to drought in 2018, the results differ between years. 

The calculated trait AREA correlated better to DIA than to WAL. Also, it behaved like DIA when correlated to developmental and yield parameters (Figure 2), indicating that DIA had a stronger impact on AREA than WAL, making it a more worthwhile target for improving lodging resistance.

Regarding yield parameters, the correlations of WAL were weaker than those for DIA. This observation pertained for all correlations between peduncle morphology and yield parameters (Figure 2B,D). Whereas a significant negative correlation for ears m^−2^ and culm diameter has been described for spring wheat [15], a second study [16] has also described a connection between the two traits in rice. In the present study, the correlation was only moderate in strength. A closer look at the positive correlations between peduncle morphology and yield parameters revealed a positive correlation with grain number per ear and grain width. In contrast, grain length was negatively correlated with DIA and AREA. Interestingly, WAL was the only peduncle morphology trait showing a significant correlation with grain yield in 2018, though it was very weak (*r* = −0.10). An increased culm thickness might therefore only be possible at the expenses of grain yield, while increasing the culm diameter can be realized without yield losses.

### 2.3. GWAS

Generally, the traits showed different prediction abilities (Table 3). Whereas the GWAS calculated with the BLUEs of DIA resulted in an average of 36.81 significant SNPs at a prediction ability of 47%, the GWAS with the WAL data did not exceed 20% prediction ability, with 27.15 significant SNPs on average. AREA, as a derived trait of DIA and WAL, performed with 40% prediction ability and 32.70 SNPs per cross-validation run on average. It is striking that the GWAS performed with a higher prediction ability for DIA and AREA in 2017, while in 2018, the prediction ability for WAL exceeded the result of the trial year 2017. For WAL, differences in GWAS prediction ability between years were not surprising due to the high impact of the environmental variance component (Table 2). Presumably, drought and the lack of heavy rainfall events and lower humidity in general led to no lodging and fewer pathogens in 2018. This lack of external influence on the culm seemed to result in a better prediction ability for WAL 2018. The difference between DIA in the single-year GWAS outcomes was not expected to be that pronounced because of its better heritability. Taking into account the differences between the outcome of the GWAS for each single year and the results of BLUEs, 2017 and 2018 need to be considered comparatively to evaluate QTL.

**Figure 3:**
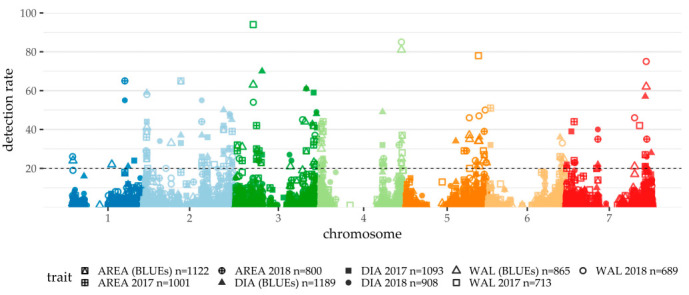
Manhattan plot summarizing cross-validated genome-wide association study (GWAS) results of peduncle morphology traits 2017 and 2018. The physical SNP positions were taken from [18]. The different point shapes and fillings differentiate the traits and years. The detection rate (DR) in 100 cross-validation runs is given on the y-axis. The dashed line indicates the threshold of DR > 20, which was used as significance threshold. Markers without any detections are not displayed in the plot.

The GWAS on trait BLUEs resulted in 22 significantly associated (DR ≥ 20) SNPs for DIA and 17 each for WAL and AREA, respectively (Tables S1 and S2, Figure 3). Most of the SNPs showed relatively small effects, while a few stuck out with more significant effects. To determine robust QTL regions, the GWAS results of each year were taken into account. Closely linked SNPs of different single-year GWAS, GWAS of BLUEs or different peduncle morphology traits were treated as shared QTL regions. This way, seven shared QTL regions were defined (Table 4), which are discussed below. All genes that are mapped in the respective QTL regions are listed in Table S3. The proposed candidate genes were selected by an analysis of recent literature in terms of already described genes and experience from former HEB-25 studies.

#### 2.3.1. QPed.shared.2H-1

The QTL region on the short arm on chromosome 2H had contrary effects on DIA and WAL. Whereas wild alleles exerted a negative effect for the DIA trait, they were linked to a positive effect on the trait WAL. Interestingly, this QTL could not be detected for DIA and AREA in 2018, hinting to a reduced impact of this QTL on DIA reduction under dry conditions, while WAL remained unaffected. This hypothesis is based on the finding that earliness for plants can be beneficial to escape summer hotness and drought [19]. As drought is thought to have a strong impact on the trial results and *PPD-H1* is the main driver in the population for the determination of the flowering time, both were taken in account to explain the findings. The responsive wild *Ppd-H1* allele would therefore adapt the plant better to drought as it occurred in Halle in 2018. Even under late drought, those plants can develop their uninfluenced habitus. For late genotypes carrying *ppd-H1*, only the traits which manifest early in the vegetation period and therefore under sufficient water supply can develop their uninfluenced habitus. Following this hypothesis, DIA may be determined in a later developmental stage and could not have developed entirely in 2018 before drought. In contrast, WAL might manifest early in the plant’s life cycle and therefore unfolded in both years.

The SNP with the highest DR for DIA and AREA is 73,564 bp away from the gene *PPD-H1 (PSEUDO-RESPONSE REGULATOR 7)*, which accelerates reproductive developmental processes when the plants are subjected to long-day conditions [20], while the distance to the SNP with the highest DR for WAL is 541,824 bp (based on BLUEs). 

The effect of *PPD-H1* bases on its role in the circadian clock [21]. Represented alleles in the HEB-25 population range from reduced response to long days introduced by the cultivated Barke allele and high sensitivity to long photoperiods as described for most wild barley accessions. The wild allele is assumed to accelerate floral development with a trade-off in growth and biomass production [14], which corresponds to the observed reduction in DIA and AREA in the present study. Therefore, *PPD-H1* is a likely candidate gene located in this QTL region controlling DIA and AREA. Due to the distance of the peak SNP from *PPD-H1* and the contrary effect direction, a different adjacent gene controlling WAL is assumed, although the low impact in family 24 (Table S1) corresponds to the only *PPD-H1* haplotype present in HEB-25 that, phenotypically, does not differ from the Barke haplotype regarding flowering time [13].

To validate *PPD-H1* (HORVU.MOREX.r2.2HG0088300.1) as a candidate gene, exome capture data (Table S4) were taken into account. The exome capture data of 21 exotic parental lines were available, and each line was compared to the estimated wild allele effects on peduncle traits of the corresponding family. The most extreme values were found for family 23 and 24 regarding all trait-year combinations found at this shared QTL (Table S1). At the physical position of 23,721,387 bp, both families carry the same SNP (adenosine, while all others have guanine). At 23,722,880 bp, only family 24 stands out with a SNP (guanine instead of thymine), which might explain the reason why family 24 contrasts even more than family 23 from the others. On other side of the effect spectrum, the effects of family 17 on DIA and AREA stand out. Also, regarding the exome capture data, family 17 differs from the others at position 23,722,022 bp with a SNP (cytosine instead of guanine). As the exome capture data reflect the strongest and most robust family effects on both sides of the spectrum, we assume that *PPD-H1* has an influence on peduncle traits.

#### 2.3.2. QPed.shared.2H-2

Compared to the remaining QTL detected in this study, this QTL had the highest impact regarding effect strength (Table S1). This QTL was robust across both years and all families. Furthermore, for all three traits, decreasing effects could be attributed to the wild allele. The phosphatidyl ethanolamine-binding protein (PEBP) encoding gene *CENTRORADIALIS* (*HvCEN)* [22] is present in this QTL region. It is positioned in the low-recombining peri-centromeric region of 2H at 523,377,523 bp [18], which corresponds to a genetic position of 57 cM. *HvCEN* is a key regulator of flowering time with pleiotropic effects on shoot traits as it promotes tillering and increases plant height [24]. *PPD-H1* and *HvCEN* show unidirectional effects on HEB-25 developmental traits with reduced effect strength of *HvCEN* compared to *PPD-H1* [14]. Interestingly, regarding the peduncle traits, the potential effect of *HvCEN* on both traits is on a higher scale and on WAL, even pointing in the opposite direction. Exome capture sequencing did not reveal obvious SNPs within the gene sequence explaining the observed phenotypes. Of course, other genes controlling the traits cannot be excluded due to the high LD in the peri-centromeric region. For instance, we found hints of the presence of AP2-like ethylene-responsive transcription factors (Table S3), which belong to one of the largest gene families, encoding plant-specific transcription factors [25]. *AP2* was causally connected to peduncle cell division and peduncle cell expansion in barley [26]. During flowering, *AP2* is involved in the modulation of the jasmonate pathway to facilitate gibberellin-promoted stem growth [26]. Unfortunately, due to the complexity of this gene family, we were not able to define specific exome capture sequences for the obtained candidate genes.

#### 2.3.3. QPed.shared.2H-3

This QTL region strongly and positively affected the DIA and AREA traits in five, while the effect in the remaining families was negligible. HORVU2Hr1G093690.1 encoding Expansin A5, a cell wall development-related protein that was proved to induce cell wall extension and cell expansion [27], was located close to the peak marker of the QTL. However, exome capture sequencing did not reveal obvious SNPs within the gene sequence explaining the observed phenotypes.

#### 2.3.4. QPed.shared.3H-1

The peri-centromeric region of 3H (centromere position: 271,947,776 bp [18]) contains the QTL region with the highest DR (up to 94 detections for WAL 2017) and positive effects on all three traits in all families except family 1. In both trial years, the effects were robust and strong. The QTL region comprises 265,569,313 bp, which makes it difficult to define a corresponding candidate for this QTL. Due to its effect strength and robustness over the years, the causative gene is valuable for peduncle optimization and advanced research. 

#### 2.3.5. QPed.shared.3H-2

This QTL region is based on a single marker (JHI_Hv50k_2016_194212), which had a DR of 61 for DIA BLUEs and 2018 each, and a DR of 29 for AREA BLUEs. Overall, the mean effect of all 25 families was moderate, but stood out for the families 20, 21, and 22. The wild alleles’ effects of these three families reached a 93.5 µm to 123.7 µm increase in DIA compared to the Barke allele. Positive effects of similar strength were only reached by QPed.shared.3H-1. For AREA, the same pattern can be seen regarding the family-specific effects, but the maximum effects did not reach the effect strength of the comparable QPed.shared.3H-1.

#### 2.3.6. QPed.shared.3H-3

This QTL was shown to exhibit significant effects, leading to a decrease in all three traits exclusively in 2017. It is suggested that the early maturity induced by drought might have impeded further peduncle growth before the effect of this QTL region would have started to influence the traits in 2018. The QTL region contains 4,457,697 bp. The *DENSO* (*sdw1*) locus corresponds to this QTL region [28]. It contains a semi-dwarfing allele often used by breeders worldwide. *DENSO* positively affects lodging resistance, yield, and heading date and increases the harvest index [28]. The gene underlying the locus is *HvGA20ox*_2_, which encodes a GA-20 oxidase, a functional gene for gibberellic acid biosynthesis [28]. Being aware of gibberellins and their involvement in plant growth and development, it is less surprising that *DENSO* had a multilevel dwarfing effect on cells, tissues, and organs in barley [29]. The effects differed in strength depending on organ and cell type. However, as leaf sizes were reduced, vascular bundles became smaller, and tracheal elements were closer packed in leaves [29]. It is likely that *DENSO* affected the peduncle morphology as well. However, exome capture sequencing did not reveal obvious SNPs within the gene sequence explaining the observed phenotypes. In the study conducted by Herzig et al. [30], there was a QTL defined for lodging (QTL-3H-11), which overlapped with QPed.shared.3H-3. This overlap, together with the estimated effects, confirmed the correlation of peduncle traits with lodging in HEB-25 (Figure 2).

#### 2.3.7. QPed.shared.4H-1

The region was already described as a major QTL for flowering time [13], and numerous pleiotropic effects on plant development and yield components have been observed in the HEB-25 population [14,31]. We observed a robust effect on WAL, while DIA and AREA were only affected in 2018. The effect strength of the individual families was very heterogeneous, with the effects of family 9 and 12 being highest for WAL with increases of 13–15 µm in both years. The region comprises 2,196,419 bp on the distal end of the short arm of chromosome 4H.

### 2.4. Impact of Plant Development on Peduncle Morphology

The strongest impact on peduncle shape could be attributed to three QTL regions (QPed.shared.2H-1, QPed.shared.2H-2, QPed.Shared.3H-1). Each of them is associated with all three traits. The effect strengths and directions in-between the peduncle traits are comparable, except for QPed.shared.2H-1, where effects on DIA and WAL are opposite. Two of the main regions (QPed.shared.2H-1, QPed.shared.2H-2) and two weaker QTL regions (QPed.shared.3H-3, QPed.shared.4H-1) coincide with major QTL, controlling the flowering time in spring barley (Table 5) [13]. This raises the question of how far peduncle morphology might indirectly be determined by flowering time. This trend is also visible in the positive correlation between HEA and DIA. However, the effect strengths of flowering time QTL and peduncle QTL are not comparable. Whereas the maximum effect on flowering time was observed for the QTL on QPed.shared.2H-1, the maximum effect on DIA was assigned to QPed.shared.2H-2. Furthermore, three major flowering time QTL were not detected for peduncle traits (QFt.HEB25-1b, QFt.HEB25-4e, QFt.HEB25-7a) [13]. Likewise, QPed.shared.3H-1 had a strong impact on peduncle traits and was not detected for HEA at all. Summed up, a more differentiated role of phytohormones controlling the flowering time, as well as conferring direct effects on cell growth and differentiation, needs to be assumed. 

In bamboo, shoot apical meristem width, height, and the total cell number are related to culm diameter [32]. Moreover, it has been shown that differences in abscisic acid, auxins, gibberellins, and jasmonates levels cause the differences between the tested bamboo accessions [32]. This indicates that alternations in plant cell walls are more likely an indirect consequence of mutations in genetic elements controlling developmental programs than a mutation directly in a gene involved in cell wall biogenesis [33]. Transferring those findings to the HEB-25 population might explain the influence of genomic regions containing major developmental genes, connected to different phytohormones, on peduncle morphology. 

## 3. Materials and Methods 

### 3.1. Plant Material and Field Trials 

The NAM population “Halle Exotic Barley 25” (HEB-25) was generated by crossing the spring barley elite cultivar Barke (*Hordeum vulgare* ssp. *vulgare*) with an assortment of 25 exotic barley accessions. The exotic founders were 24 *H. vulgare* ssp. *spontaneum* accession and 1 Tibetan *H. vulgare* ssp. *agriocrithon*. The accessions were chosen from the work described by Badr et al. [13], as it covers a major part of genetic diversity present in the domestication area of barley, the Fertile Crescent. After backcrossing the resulting F1 plants to Barke and 3 rounds of selfing by means of single seed descent, 1420 BC1S3 lines, divided in 25 families each containing up to 75 lines, were propagated as individual bulks [12]. The population is mainly composed of 2-rowed barley, as is the elite founder Barke. Only family 24 partly consists of 6-rowed phenotypes.

The field trials were conducted in 2017 and 2018 with the HEB-25 generations BC1S3:10 and BC1S3:11. They were performed at the Kühnfeld Experimental Station (51°29′46.47″ N; 11°59′41.81″ E) in a partially replicated design with the reference line Barke growing in 11 plots (2017) or 16 plots (2018). Each line grew in a single plot each year, with 2 rows in 2017 and 3 rows in 2018, respectively. The rows amounted to 140 cm in length, containing 50 seeds each, while the distance between rows and plots corresponded to 20 cm and 50 cm, respectively. For detailed information about field trial management, see Table S5.

### 3.2. Phenotyping

**Figure 4:**
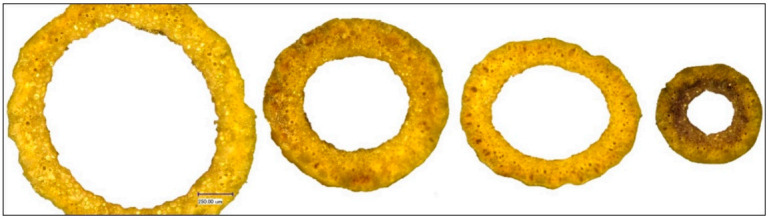
Diameter of selected HEB-25 lines and Barke as reference. From left to right: HEB_03_142, Barke, HEB_24_188, HEB_08_016.

**Figure 5:**
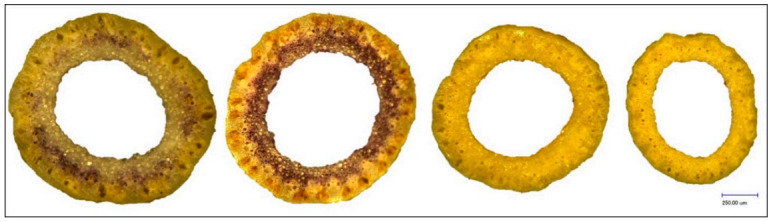
Thickness of culm tissues of selected HEB-25 lines and Barke as reference. From left to right: HEB_23_037, HEB_20_017, Barke, HEB_14_005.

At maturity, 5 main tillers per plot, each taken from a different random plant (=5 biological replicates), were harvested. Of each culm, a cross-section directly under the collar of the spike was prepared and the diameter (DIA) and thickness of culm tissues (WAL) were measured in micrometers with the digital microscope Keyence VHX-500F with standard zoom lens VH-Z20R (×150) (Figure 4 and Figure 5). Each trait was measured repeatedly for each cross-section in different positions (Figure S2). 

Out of the 2 measured traits, the surface of the cross-section was calculated (AREA).

$$AREA=\;\pi \;{\left(\frac{DIA}{2}\right)}^{2}-\;\pi \;{\left(\frac{DIA}{2}-WAL\right)}^{2}$$

The mean of the 5 tiller measurements was taken for further analyses.

### 3.3. Statistics and Significance Tests

Basic descriptive statistics and t-tests between years were calculated in SAS 9.4 (SAS Institute Inc., Cary, NC, USA). The coefficient of variation (CV) was determined across the whole population by dividing the standard deviation by the mean value. Pearson correlations were calculated using the “cor” function in R 3.4.3 (R Development Core Team, Vienna, Austria). 

In order to make a statement about the ratio of total genotypic variance to total phenotypic variance, the broad-sense heritability (*H*^2^) was calculated across the 2 trial years. For this, variance components were estimated with SAS PROC VARCOMP based on a linear model assuming random effects for genotype and environment:

yij = µ + gi + ej + εij

$$H^{2}=\;\frac{V_{G}}{V_{G}+\frac{V_{R}}{2}}$$

where *V_G_* = genotypic variance, *VE* = environmental variance, *V_R_* = residual variance, yij = trait value of the *i*-th genotype in the j-th environment, µ = intercept, gi = random effect of the *i*-th genotype, ej = random effect of the j-th environment (=trial year), and εij = residual.

To estimate BLUEs for each line across both years, the abovementioned model was applied, assuming fixed genotype effects in a linear mixed model (SAS PROC HPMIXED).

### 3.4. Genome-Wide Association Study (GWAS)

GWAS was conducted for each single year separately, as well as for trait BLUEs. GWAS was based on 50k Illumina Infinium iSelect SNP Array [11] genotyping, applied at TraitGenetics (Gatersleben, Germany). An IBS matrix with flanking marker imputation of missing genotype scores was derived, containing 32,995 SNP markers [15]. To achieve robust QTL detection with corresponding estimation of the family-specific effects, cross-validation and subsequent accumulation of estimated marker effects around a central peak marker [16] was performed. The GWAS was based on multiple linear regression with SAS PROC HPREG, selecting those SNPs out of all 32,995 SNPs accounting for the best model based on stepwise forward-backward regression with SNPs entering or leaving the model at each step if the p-value calculated for the marginal F-test was <0.001. Ensuring robust results, a 5-fold cross-validation was repeated 20 times. Thus, 100 cross-validation runs were performed. For each run, a training set covering 80% of random HEB-Lines of each family was created, and significant markers and their estimated effects were defined via GWAS. The other 20% of HEB lines were used for validation in the form of a calculated squared Pearson product-moment correlation between observed and predicted phenotypes of validation set, which represents the cross-validated proportion of explained genotypic variance and is referred to as the prediction ability. The mean prediction ability in 100 cross-validation runs (20 times fivefold cross-validations) was taken as the final record. 

To define a QTL as robust, the SNP had to be detected in more than 20 out of the 100 cross-validation runs, which is referred to as detection rate (DR). The threshold was set after a permutation test based on 3 shuffled genotype-phenotype matrices used for the abovementioned cross-validation procedures (≙300 cross-validation runs). Based on the obtained results, we observed a detection rate of 20 as the 99.99th percentile (that means 99.99% of markers have less detections). If the observed detection rate from the original data exceeded this threshold, we declared the presence of a significant marker trait association.

Family-specific effects were determined by applying the cumulation method [16], accumulating SNP marker effects around a central peak marker with highest DR in a 26 cM-sized region for each of the 25 donors and cross-validation runs. The average of all cross-validation runs was then defined as a family-specific effect.

Physical map positions of the barley Infinium iSelect 50k chip were taken from [11]. The genetic map was based on the map for the 9k chip presented by the authors of [12], based on [17]. In the case of 4722 SNPs that were shared between the 9k and the 50k chip, the genetic positions of the 50k markers were taken from [12]. The genetic positions of the remaining markers were estimated based on the genetic positions of physically adjacent markers. 

### 3.5. Exome Capture Sequencing

Exome capture reads first passed a quality enrichment process to remove sequence adapter remains and low-quality sequences using TrimGalore (https://github.com/FelixKrueger/TrimGalore). Subsequently, quality improved reads were aligned with BWA mem [18] to the barley genome reference sequence (version 2) of cultivar Morex [19]. From the constructed alignments, read duplicates were removed by samtools [20]. We then merged the 21 wild barleys and Barke into a single BAM file. This file was used for SNP calling using Freebayes [21] applying parameter ‘--min-alternate-count 3 --min-alternate-fraction 0.05 --min-coverage 10 --no-complex --dont-left-align-indels --no-population-priors’. High-quality SNPs were filtered by bcftools [14], in which each SNP was required to have a vcf quality score above 1000. The respective candidate genes (HORVU.MOREX.r2.2HG0088300.1, HORVU.MOREX.r2.2HG0136910.1, HORVU.MOREX.r2.2HG0153910.1, HORVU.MOREX.r2.3HG0256590.1) were extracted using bedtools ‘intersect’ [22]. The matrix of filtered SNPs from the HEB-25 wild barley donors are available as Supplementary Table S4.

## 4. Conclusions

It was possible to gain new insights into the QTL regions involved in controlling peduncle morphology. A connection between lodging and peduncle traits was shown, while a direct impact on grain yield could not be observed. Three QTL regions on 2H and 3H stood out through robust and strong effects on peduncle morphology traits. Although flowering initiation coincided with peduncle elongation, the effects of QTL controlling these traits were shown to be less conforming than expected. This might have been caused by the phytohormones differentially affecting cell growth as well as flowering time. QPed.shared.3H-1 (47.7–51.8 cM) showed strong positive effects on the peduncle traits but none on flowering time. However, due to high LD in the peri-centromeric QTL region, further studies need to be performed to identify the underlying causative gene. Beneficial wild alleles of this gene might be a promising target for introgression into elite barley to increase the lodging resistance and/or amount of harvested straw while keeping other favorable properties of plants unaffected.

## Figures and Tables

**Table 1:** Descriptive statistics for each year and best linear unbiased estimates (BLUEs) across environments (DIA/WAL [µm], AREA [µm^2^]).

Trait ^a^	N ^b^	Mean ^c^	SD ^d^	Min ^e^	Max ^f^	CV ^g^	Barke ^h^
DIA17	1232	1202.82	141.08	776.00	1640.91	0.12	1239.35
DIA18	1343	1204.25	137.89	833.50	1931.10	0.11	1309.98
DIA	1411	1204.33	126.87	841.76	1876.26	0.11	1274.66
WAL17	1232	279.01	24.01	179.96	385.95	0.09	270.66
WAL18	1343	247.32	19.98	178.74	322.83	0.08	238.83
WAL	1411	263.16	18.84	179.35	338.69	0.07	254.74
AREA17	1232	8.14 × 10^5^	1.57 × 10^5^	3.78 × 10^5^	1.35 × 10^6^	0.19	8.24 × 10^5^
AREA18	1343	7.46 × 10^5^	1.32 × 10^5^	4.12 × 10^5^	1.37 × 10^6^	0.18	8.04 × 10^5^
AREA	1411	7.81 × 10^5^	1.29 × 10^5^	4.04 × 10^5^	1.29 × 10^6^	0.16	8.16 × 10^5^

^a^ Traits separated by years and trait BLUEs. ^b^ Number of observations. ^c^ Arithmetic mean. ^d^ Standard deviation. ^e^ Minimum. ^f^ Maximum. ^g^ Coefficient of variation across population. ^h^ Mean of reference variety Barke.

**Table 2:** Broad-sense heritability across all genotypes of the traits DIA, WAL, and AREA with respective variance components.

Trait	V_g_^a^	V_e_^b^	V_r_^c^	H^2 d^
DIA	57.8	0.0	42.2	73.3
WAL	17.4	51.1	31.4	52.6
AREA	45.2	10.4	44.4	67.0

^a^ Genotypic variance component. ^b^ Environmental variance component. ^c^ Error variance component. ^d^ Broad-sense heritability (%).

**Table 3:** Mean number of significant single nucleotide polymorphisms (SNPs) and cross-validated prediction ability obtained by those SNPs, for the traits DIA, WAL, and AREA. Data for single trial years and BLUEs.

Trait	Prediction Ability	⌀Number of Sig. SNPs
DIA	0.47	36.81
DIA17	0.45	31.67
DIA18	0.28	25.75
WAL	0.20	27.15
WAL17	0.10	21.41
WAL18	0.21	24.42
AREA17	0.33	28.46
AREA18	0.28	23.35
AREA	0.40	32.70

**Table 4:** Mean wild allele effects of peak markers (DR > 20) of selected quantitative trait locus (QTL) regions.

QTL Region				DIA [µm]			WAL [µm]			AREA [µm^2^]			Candidate Gene
*shared-QTL*	*chromosome*	*Position ^a^*		*2017*	*2018*	*BLUEs*	*2017*	*2018*	*BLUEs*	*2017*	*2018*	*BLUEs*	
QPed.shared.2H-1	2H	18.3–26.8 cM	21,508,891–29,254,219 bp	−101.5	-	−67.5	5.6	6.5	5.5	−8.3 × 10^4^	-	−4.4 × 10^4^	*PPD-H1* [20]
QPed.shared.2H-2	2H	55.5–60.8 cM	164,050,763–518,354,808 bp	−181.6	−153.7	−168.0	−10.2	−7.5	−9.5	−1.8 × 10^5^	−1.4 × 10^5^	−1.6 × 10^5^	*HvCEN* [22]
QPed.shared.2H-3	2H	82.0 cM	586,582,931–586,594,551 bp	11.3	-	16.3	-	-	-	−1.3 × 10^4^	-	1.0 × 10^4^	
QPed.shared.3H-1	3H	47.7–51.8 cM	128,830,583–394,780,497 bp	72.7	100.9	91.1	10.5	14.4	14.2	8.8 × 10^4^	1.2 × 10^5^	1.1 × 10^5^	
QPed.shared.3H-2	3H	71.5 cM	517,985,664 bp	-	17.0	16.3	-	-	-	-	-	2.1 × 10^4^	
QPed.shared.3H-3	3H	106.6–109.5 cM	569,528,666–573,503,074 bp	−50.3	-	-	−8.8	-	−2.0	−6.0 × 10^4^	-	−2.4 × 10^4^	*HvGA20ox_2_* [23]
QPed.shared.4H-1	4H	0.9–2.1 cM	393,618–3,342,910 bp	-	25.8	-	5.1	8.1	6.3	-	3.7 × 10^4^	2.5 × 10^4^	

^a^ Interval between most extreme positions of significant markers found inside shared QTL.

**Table 5:** Effect comparison of heading (HEA) QTL and corresponding peduncle QTL regions.

QTL	HEA ^a^	WAL ^b^	DIA ^b^	AREA ^b^
QFt.HEB25-1b ^a^	−1.4	-	-	-
QPed.shared.2H-1	−9.5	5.5	−67.5	−4.4 × 10^4^
QPed.shared.2H-2	−3.0	−9.5	−168.0	−1.6 × 10^5^
QPed.shared.3H-1	-	14.2	91.1	1.1 × 10^5^
QPed.shared.3H-3	−3.1	−2.0	−22.3	−2.4 × 10^4^
QPed.shared.4H-1	3.2	6.3	25.2	2.5 × 10^4^
QFt.HEB25-4e ^a^	2.2	-	-	-
QFt.HEB25-5d ^a^	3.8	−1.3	−22.7	-
QFt.HEB25-7a ^a^	4.1	-	-	-

^a^ taken from Maurer et al. [13]. ^b^ based on BLUEs.

## Data Availability

All data is included in Supplementary Files and available upon request.

## Supplementary Materials

The following are available online at https://www.mdpi.com/2223-7747/10/1/10/s1, Figure S1: Daily precipitation and air temperature-weather station Kühnfeld Halle; Figure S2: Cross-section measuring example for WAL and DIA; Table S1: Family-specific effects for QTL with peak markers exceeding a detection rate >20 out of 100 cross-validation runs; Table S2: GWAS estimates for all markers obtained by cross-validated GWAS of peduncle morphology traits; Table S3: List of all predicted genes in respective QTL regions based on BARLEYMAP; Table S4: Exome Capture SNP Data; Table S5: Field trial management.
