# Supplementary figures and images for: Genomic Dissection of Peduncle Morphology in Barley through Nested Association Mapping
Source: Plants (Basel). 2020 Dec 23;10(1):10. doi: 10.3390/plants10010010 (PMC7823623; doi:10.3390/plants10010010)

Figure S1. Daily precipitation and air temperature - weather station Kühnfeld Halle

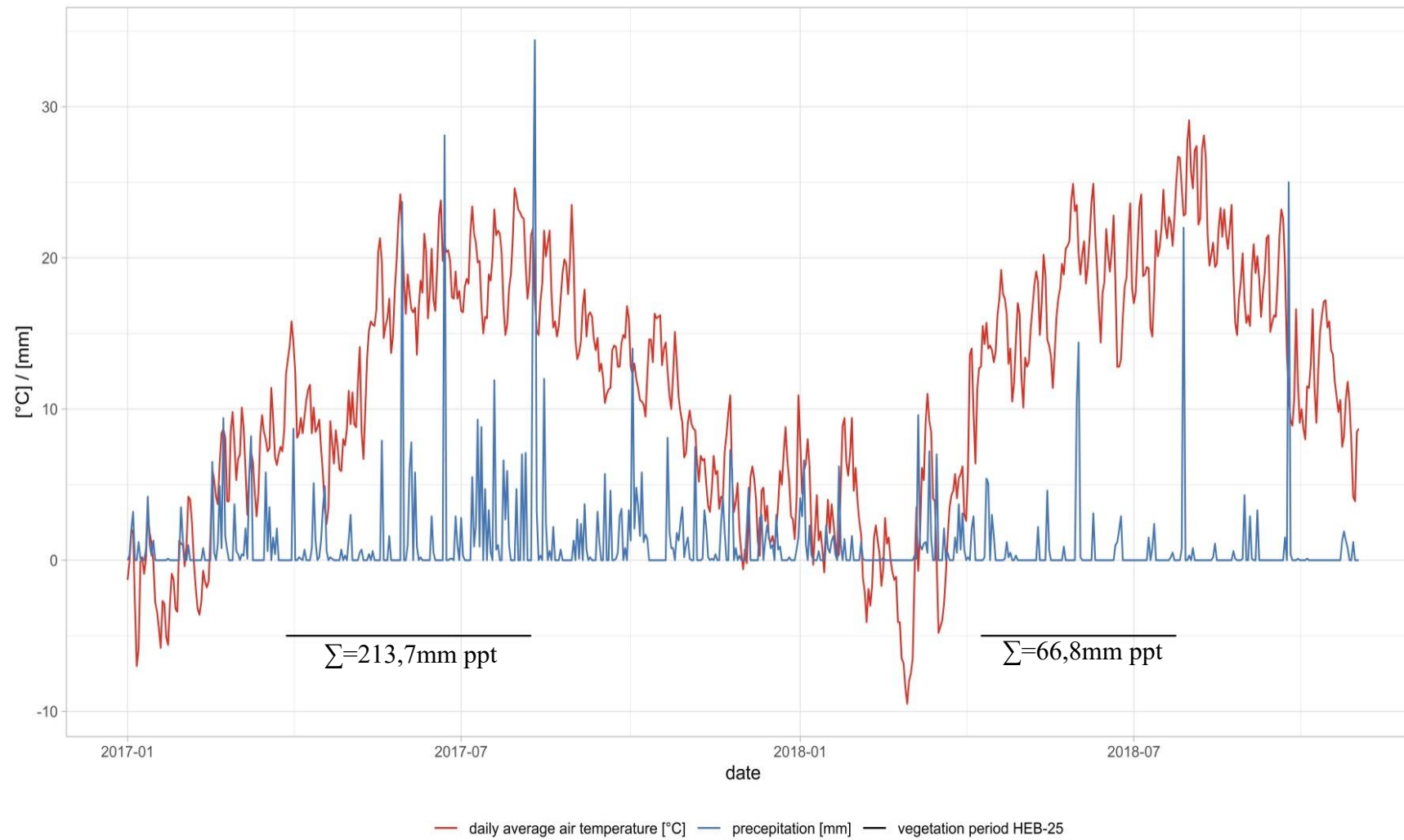

Supplement: Supplementary file 1 [file plants-10-00010-s001.zip › Figure S1.pdf]

Figure S2. Cross-section measuring example for WAL and DIA

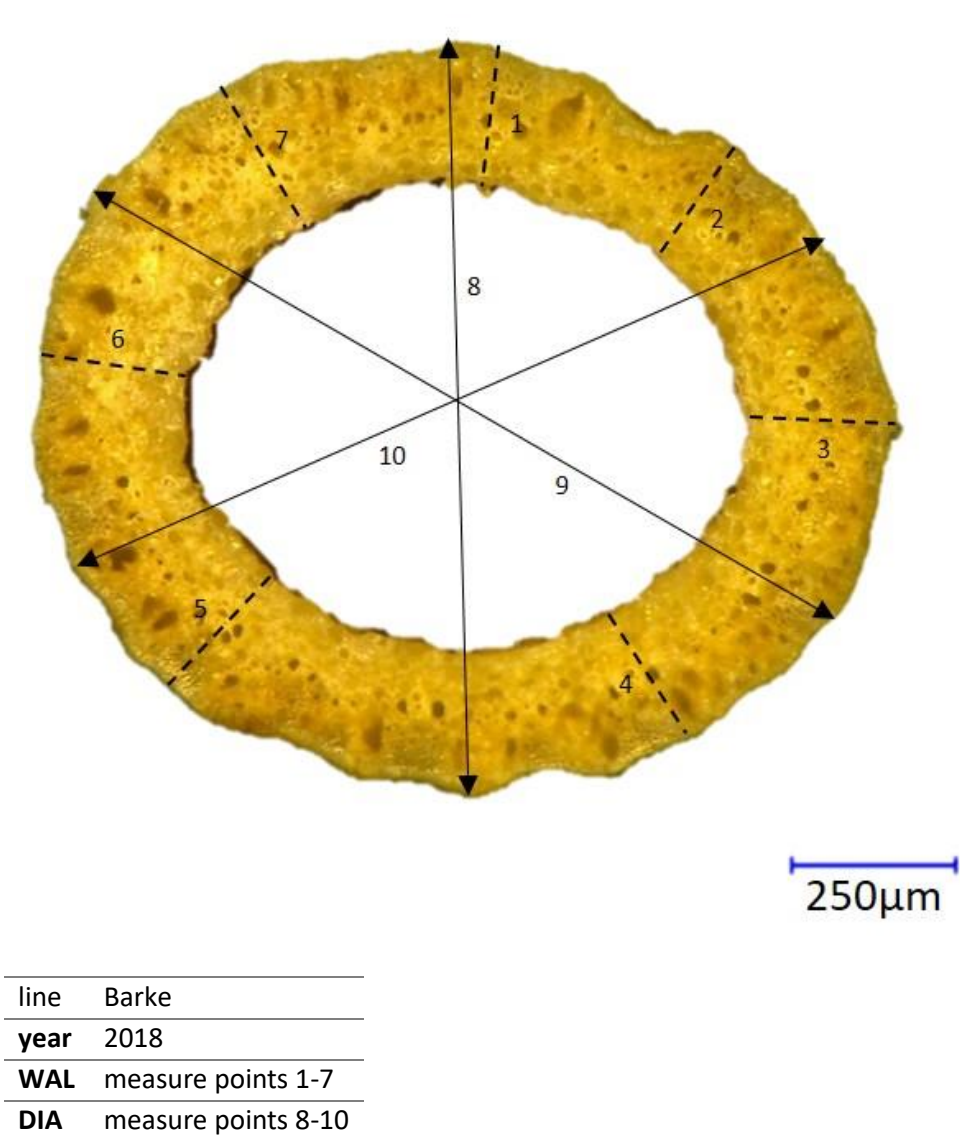

Supplement: Supplementary file 1 [file plants-10-00010-s001.zip › Figure S2.pdf]
